# Keratin 8 is a potential self-antigen in the coronary artery disease immunopeptidome: A translational approach

**DOI:** 10.1371/journal.pone.0213025

**Published:** 2019-02-27

**Authors:** Peter M. Mihailovic, Wai Man Lio, Romana Herscovici, Kuang-Yuh Chyu, Juliana Yano, Xiaoning Zhao, Jianchang Zhou, Bo Zhou, Michael R. Freeman, Wei Yang, Prediman K. Shah, Bojan Cercek, Paul C. Dimayuga

**Affiliations:** 1 Oppenheimer Atherosclerosis Research Center, Smidt Heart Institute, Cedars-Sinai Medical Center, Los Angeles, California, United States of America; 2 Faculty of Medicine, University of Ljubljana, Ljubljana, Slovenia; 3 Division of Cancer Biology and Therapeutics, Department of Biomedical Sciences, Cedars-Sinai Medical Center, Los Angeles, California, United States of America; 4 Department of Surgery, Cedars-Sinai Medical Center, Los Angeles, California, United States of America; Centro Cardiologico Monzino, ITALY

## Abstract

**Background:**

Inflammation is an important risk factor in atherosclerosis, the underlying cause of coronary artery disease (CAD). Unresolved inflammation may result in maladaptive immune responses and lead to immune reactivity to self-antigens. We hypothesized that inflammation in CAD patients would manifest in immune reactivity to self-antigens detectable in soluble HLA-I/peptide complexes in the plasma.

**Methods:**

Soluble HLA-I/peptide complexes were immuno-precipitated from plasma of male acute coronary syndrome (ACS) patients or age-matched controls and eluted peptides were subjected to mass spectrometry to generate the immunopeptidome. Self-peptides were ranked according to frequency and signal intensity, then mouse homologs of selected peptides were used to test immunologic recall in spleens of male apoE-/- mice fed either normal chow or high fat diet. The peptide detected with highest frequency in patient plasma samples and provoked T cell responses in mouse studies was selected for use as a self-antigen to stimulate CAD patient peripheral blood mononuclear cells (PBMCs).

**Results:**

The immunopeptidome profile identified self-peptides unique to the CAD patients. The mouse homologs tested showed immune responses in apoE-/- mice. Keratin 8 was selected for further study in patient PBMCs which elicited T Effector cell responses in CAD patients compared to controls, associated with reduced PD-1 mRNA expression.

**Conclusion:**

An immunopeptidomic strategy to search for self-antigens potentially involved in CAD identified Keratin 8. Self-reactive immune response to Keratin 8 may be an important factor in the inflammatory response in CAD.

## Introduction

Inflammation plays an important role in atherosclerosis, the underlying cause of coronary artery disease (CAD) [1]. It is a significant risk factor for a cardiovascular event underscored by the outcome of the CANTOS trial [2], where residual inflammatory risk [3,4] was targeted for therapy. Clinical biomarkers of inflammation in CAD do not completely disclose the fundamental characteristics of the process nor do they reveal the pathways involved.

Unresolved inflammation by the innate immune response will have effects on the adaptive immune system [5], as reported in CAD patients [6]. The underlying simmering inflammation in CAD patients [2] may result in maladaptation of the adaptive immune responses toward self-antigens [7]. Investigations of potential self-antigens in CAD have focused primarily on LDL [8,9], or apoB-100 specifically [10–13]. However, the multi-factorial nature of CAD makes it unlikely that self-antigens in CAD would be limited to LDL-derived antigens. Self-antigens become unintended targets of the maladaptive immune response in unresolved inflammatory conditions. Self-antigens are processed through proteasomal degradation of intracellular proteins as part of normal cellular homeostasis and presented as self-peptides on MHC-I molecules on the cell surface [14,15]. MHC-I molecules present self-peptides to CD8+ T cells, which normally ignore or are tolerant to these self-antigens. This process is considered to be a key element of immune surveillance [15]. However, under conditions of persistent inflammation in diseased states, response to self-antigens is altered and is postulated to contribute to the development of autoimmunity [16]. Psoriasis is one such autoimmune condition that is associated with increased risk of cardiovascular events [17] and a T cell-reactive self-antigen in psoriasis has been identified [18]. Using the mouse homolog of the self-antigen, we recently reported its potential as a T cell self-antigen in atherosclerosis [19], linking an autoimmune self-antigen and atherosclerosis. We were thus compelled to develop a method to identify and investigate other potential self-antigens in the context of CAD.

Shedding of the MHC-I/peptide complex has been exploited to identify potential self-reactive antigens in disease [20,21]. We therefore hypothesized that self-antigens involved in CAD are present as peptides complexed to soluble MHC-I shed during the disease process and that this can be potentially exploited to generate an immune-peptidomic profile of self-antigens in CAD.

We used immuno-precipitation (IP) of soluble MHC-I/peptide complexes from plasma samples [20,21] of acute coronary syndrome (ACS) patients and subjected the peptides to ultra-performance liquid chromatography-tandem mass spectrometry (UPLC-MS/MS) to identify potential self-antigens. ACS patient samples are suitable for our study because these patients categorically have CAD. The antigenic potential of the identified self-peptides to provoke Effector Memory (EM) T cell response was tested using mouse homologs in apoE-/- mice. EM T cell response in mice was used based on the reports that correlated T Effector Memory cells with CAD [6], and that the highest presence of the cell type in thin-cap fibroatheroma and ruptured plaques in patients are T Effector Memory cells [22].

We then explored the translational relevance of our findings by testing the T Effector Memory response to peptide stimulation of PBMCs from CAD patients with one of the identified self-antigens–Keratin 8. Our study provides a strategy to identify and test novel self-antigens that may be involved in CAD. The results may begin to shed new light on the pathways of inflammatory signaling that contributes to CAD and have the potential to develop new paradigms in understanding the disease.

## Materials and methods

### Patient plasma

Research was conducted according to the principles expressed in the Declaration of Helsinki. Plasma samples were obtained from frozen aliquots that remained from a previously completed IRB-approved study called AZACS (AZythromycin in Acute Coronary Syndrome) [23]. Samples from 5 male patients aged 55–70 years old were randomly selected and used under an IRB approved protocol (Pro00034283), which limited use of patient data for this study to age and sex. Patient demographic and characteristics have been previously reported [23]. Plasma samples from 5 age-matched male self-reported control volunteers were purchased (Innovative Research).

### Immuno-precipitation

One ml of plasma was subjected to immuno-precipitation (IP) using the capture antibody against soluble HLA-A, -B, and -C (clone W6/32) coupled to agarose beads using a commercially available kit (AminoLink Plus Coupling, Thermo Fisher). Bead conjugated antibody was then added to plasma diluted 10x in TBS buffer with 0.01% PPS Silent Surfactant (Expedion) and rotated for 18 hours in 4°C. After IP, samples were subjected to elution according to the manufacturer’s instructions. An aliquot of the eluate was used for Western blot analysis to confirm the presence of the immuno-precipitated HLA proteins (clone EP1395Y). The samples were heat denatured at 95°C for 10 minutes, cooled, and peptides were separated from the rest of the IP eluate using size exclusion centrifugation columns cut-off at 3kD (Amicon). Western blotting was performed to confirm separation of the filtrate containing the peptides from the concentrate.

### UPLC-MS/MS

The 3kD fraction, corresponding to the peptide samples were then subjected to UPLC- MS/MS analysis at the Cedars-Sinai Mass Spectrometry and Biomarker Discovery Core Lab. Peptides were desalted by C_18_-StageTips [24], concentrated in a Speed Vac concentrator, and reconstituted in 25 μL 0.2% formic acid. Subsequently, 10 μL peptide solution was injected and loaded on a trap column (75 μm × 2 cm, C_18_), separated on an EASY-Spray analytical column (PepMapTM RSLC C18, 2 μm, 100Å, 50 μm x 15 cm), and analyzed by an LTQ Orbitrap Elite hybrid mass spectrometer (Thermo Fisher) operated in the positive ion mode essentially as described [25]. Mass spectra were acquired in a data-dependent manner, with automatic switching between MS and MS/MS scans. In MS scans, the lock mass at *m/z* 445.120025 was applied to provide internal mass calibration [26]. For MS/MS scans with higher sensitivity, up to 20 most intense peaks with charge state ≥2 were automatically selected for fragmentation by rapid collisional-induced dissociation (rCID). The acquired MS data was searched against the Uniprot_Human database (released on 02/20/2014, containing 88,647 sequences) using the Andromeda algorithm [27] in the MaxQuant (v1.3.0.5) environment [28]. The MS/MS peaks were deisotoped and searched using a 0.5 Da mass tolerance. Only peptide with a false discovery rate (FDR) of ≤1% were accepted.

### CAD patient peptides

Identified peptides unique to the patient cohort were ranked according to frequency of occurrence among the patients. In addition, patient-unique peptides found in single patients were ranked according to signal intensity. The 3 peptides highest ranked in frequency were selected for further studies. The selected peptide sequences were then used to search for corresponding mouse homologs using the BLAST search engine (NCBI). The selected homologous sequence in the mouse protein was then flanked on each side (amino and carboxy ends) with the corresponding 10 amino acids for that protein as reported by the BLAST output result, upstream and downstream respectively. If the peptide was located at one of the terminals, then peptide extension was done on one side only. It was postulated that flanking the peptides would reduce the chance of low MHC-binding because of potential differences in the binding properties of mouse and human epitopes.

### Predicted MHC-I binding

To assess the potential binding of the homologous mouse peptides to mouse MHC-I, the peptide sequences were tested in silico for predicted MHC-I binding scores using the IEDB epitope analysis software [29–33] for H2-D^b^ and H2-K^b^ mouse alleles that are found in the C57Bl6 strain. Analysis was performed assuming an optimized epitope length of 9-mers for H2-D^b^ and 8-mers for H2-K^b^ [34]. A score at or above the 20^th^ percentile rank (i.e. 0.1–20 percentile rank) was considered to have potential binding to mouse MHC-I and were selected for the studies.

### Animals

The study was carried out in strict accordance with the protocols approved by the Institutional Animal Care and Use Committee of Cedars-Sinai Medical Center (IACUC006000 and IACUC006703). Male apoE-/- mice were purchased from Jackson Lab. Male apoE-/-FoxP3-GFP mice were used to assess GFP+ FoxP3 regulatory T cells [19,35]. One group of mice was fed normal chow or high fat diet *ad lib* consisting of 0.15% cholesterol, 21% fat (TD.88137, Envigo) for 6 weeks starting at 7 weeks of age and euthanized at 13 weeks of age. Another group was maintained on normal chow and subjected to myocardial infarction (MI) at 13 weeks of age and euthanized at 19 weeks of age, 6 weeks after the MI. Mice subjected to either sham or MI surgery were injected with NSAID just prior to surgical procedure. For the MI surgery, a left thoracotomy was performed under injectable anesthesia on mice with tracheal intubation and the heart was visualized between the fourth and fifth ribs. The left anterior descending coronary artery was permanently ligated and the infarct was confirmed by blanching of the tissue. Sham surgery involved identical thoracotomy but no arterial ligation. The incision was closed with sutures, analgesic was administered, and the mice observed for recovery under a heat lamp. Analgesia was again administered 12 hours post-surgery. Echocardiography under inhalation anesthesia was performed using a Vevo 770 apparatus one day prior to surgery and one day prior to euthanasia.

### In vitro stimulation of splenocytes with peptides

Selected peptides were synthesized (LifeTein) at >95% purity. Spleens from mice were collected at euthanasia and the splenocytes were isolated after RBC lysis. Cells were placed in culture medium at a density of 1x10^6^/ml, stimulated with peptides at 20μg/ml or vehicle as control and incubated for 24 hours in a 37°C / 5% CO_2_ incubator in RPMI medium (Thermo Fisher) with 10% Heat Inactivated FBS (Omega Scientific), antibiotic-antimycotic (Gibco), and β-mercaptoethanol (Sigma). Harvested cells were washed in PBS and stained for viability (LIVE/DEAD Fixable Violet stain) for flow cytometric analysis and using the following antibodies for T Effector/Central Memory cells: CD4, CD8b, CD44, CD62L (eBioscience). FoxP3 was detected by GFP fluorescence. For cells aliquoted for intracellular cytokine staining, Monensin was added for the last 4 hours of culture. Antibodies for IFN-γ and IL-10 were used for intracellular cytokine staining after fixation and permeabilization of cells. Isotypes were used as controls. Color compensation was performed using non-stained and single-stained cells.

### Human PBMC

CAD patients included ACS and stable CAD. ACS patient peripheral blood mononuclear cells (PBMCs) were isolated from blood collected within 72 hours of admission to the Cedars-Sinai Coronary Intensive Care Unit for ST-elevation myocardial infarction (STEMI) and non-ST-elevation myocardial infarction (NSTEMI). Written consent from patients were obtained under the protocol Pro48880 approved by the Cedars-Sinai Medical Center IRB. Exclusions were inability to give informed consent, age less than 18 years old, active cancer treated with chemotherapy or radiation, patients taking immune-suppressive drugs, and pregnant women. Stable CAD patient PBMCs were isolated from blood collected on the day of angiography, written consent obtained under the approved IRB protocol Pro50839 with the same exclusions. Consented data use was limited to age, sex, LDL levels and use/non-use of cholesterol-lowering medication. PBMCs were isolated by Ficoll gradient centrifugation and cryo-preserved using standard techniques. Cryo-preserved self-reported Control PBMCs were purchased (Immunospot). PBMC donor subject characteristics are found in Table 1. All cells were stored in liquid nitrogen until experiments were performed.

**Table 1:** Human subject characteristics.

	Control (n = 15)	CAD (N = 17) (Stable CAD N = 7/ACS N = 10)
Mean Age	58.7	67.4 (81.4/59.3)
Male (%)	70	82 (90/80)
Mean LDLc (mmol/L)*	NA	2.49 (2.11/2.74)
Cholesterol-lowering (%)†	NA	65 (100/40)

NA = Not available

*values for ACS obtained at enrollment, values for Stable CAD extracted from chart history

†confirmed use of cholesterol-lowering medication.

### Peptide stimulation of human PBMC

Cryo-preserved cells were thawed and rinsed with anti-aggregate solution (Immunospot). Cells were then plated at a density of 3x10^6^/ml in RPMI 1640 medium supplemented with 10% heat inactivated pooled human serum. Cells were stimulated with 20μg/ml Keratin 8 peptide (LifeTein). Cells treated with 0.5x Cell Stimulation Cocktail (Thermo Fisher) containing PMA and ionomycin served as positive control. Cells without stimulation served as negative control. Forty-eight hours after plating, medium was replenished with the addition of 1/3 the original volume. Cells were then collected 72 hours after plating and processed for fluorescent staining for flow cytometry using the following antibodies: CD3, CD4, CD8, CD45RA, CD45RO, CD62L, and CCR7. Cell viability was assessed using LIVE/DEAD Fixable Violet stain. Unstained cells were used as control for auto-fluorescence, single-stained cells were used for fluorescent compensation, isotypes served as controls. T Effector cells were gated on CD62L(-) CCR7(-) cells and subtyped using CD45RA and CD45RO [36–38]. The results are expressed as Response Index calculated as:

$$\frac{\left(\%Peptide\;stimulation-\%No\;stimulation\right)}{\%Positive\;control}\times 100$$

The data are presented as such to control for individual differences among the samples in baseline levels and maximal stimulation response.

ELISA (Abcam) was performed to determine IFN-γ and IL-10 release in conditioned medium collected from the cultured PBMCs. For samples with a sufficient number of cells, the same cell density was plated with identical treatment as above for RNA studies. At cell collection after 72 hours of culture, total RNA was isolated using TRIzol (Thermo Fisher). Samples were then subjected to qRT-PCR with SYBR green and a primer pair for human PD-1. Cyclophilin A served as reference gene. Results are expressed as fold-change relative to non-treated cells of each sample using the ^ΔΔ^Ct method.

### Statistics

All data were tested for Normality of distribution and are reported as mean ± SD. Data for apoE-/- splenocyte stimulation that were normally distributed were analyzed using ANOVA followed by Dunnett multiple comparisons test with no peptide as control. Splenocyte data not normally distributed was analyzed using Kruskal-Wallis followed by Dunn’s multiple comparisons test with no peptide as control. Data for human PBMC that were normally distributed were analyzed using unpaired t-test. Data not normally distributed were analyzed using Mann-Whitney test. Significance was set at P<0.05.

## Results

### Immune precipitation and peptide elution from soluble class I HLA

**Fig 1:**
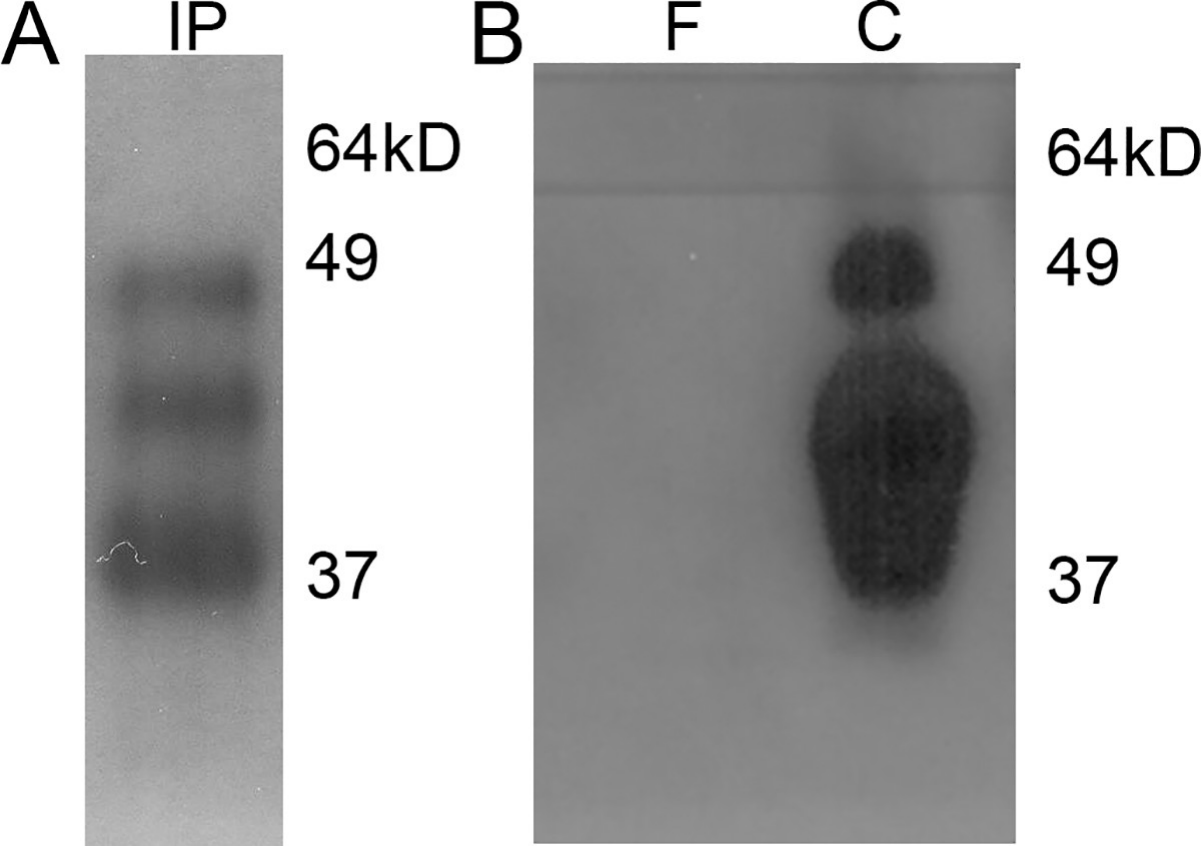
Immuno-precipitation of soluble HLA-I/peptide complexes. (A) Representative Western blot of immuno-precipitated (IP) soluble HLA-I/peptide complex eluted from agarose beads conjugated with anti-HLA-I antibody. (B) Representative Western blot of filtrate (F) and concentrate (C) fractions of the complexes after heat denaturation and 3kD size-exclusion centrifugation.

Agarose beads conjugated with the anti-HLA antibody and the captured soluble HLA/peptide complexes from plasma samples were subjected to elution to release immune-precipitated complexes. An aliquot of the eluate was then used in Western blots, which confirmed the presence of the immune-precipitate (Fig 1A). The peptides were then released from the complex using heat denaturation and subjected to size-exclusion centrifugation in 3kD cut-off columns. An aliquot of the 3kD cut-off filtrate containing the peptides and the concentrate containing the immune-precipitated HLA proteins were assessed using Western blots which confirmed separation of the lower molecular-sized peptides from the HLA proteins (Fig 1B). The peptides in the filtrate were then subjected to UPLC MS/MS analysis.

### UPLC MS/MS identifies potential self-reactive antigens in patient samples

**Fig 2:**
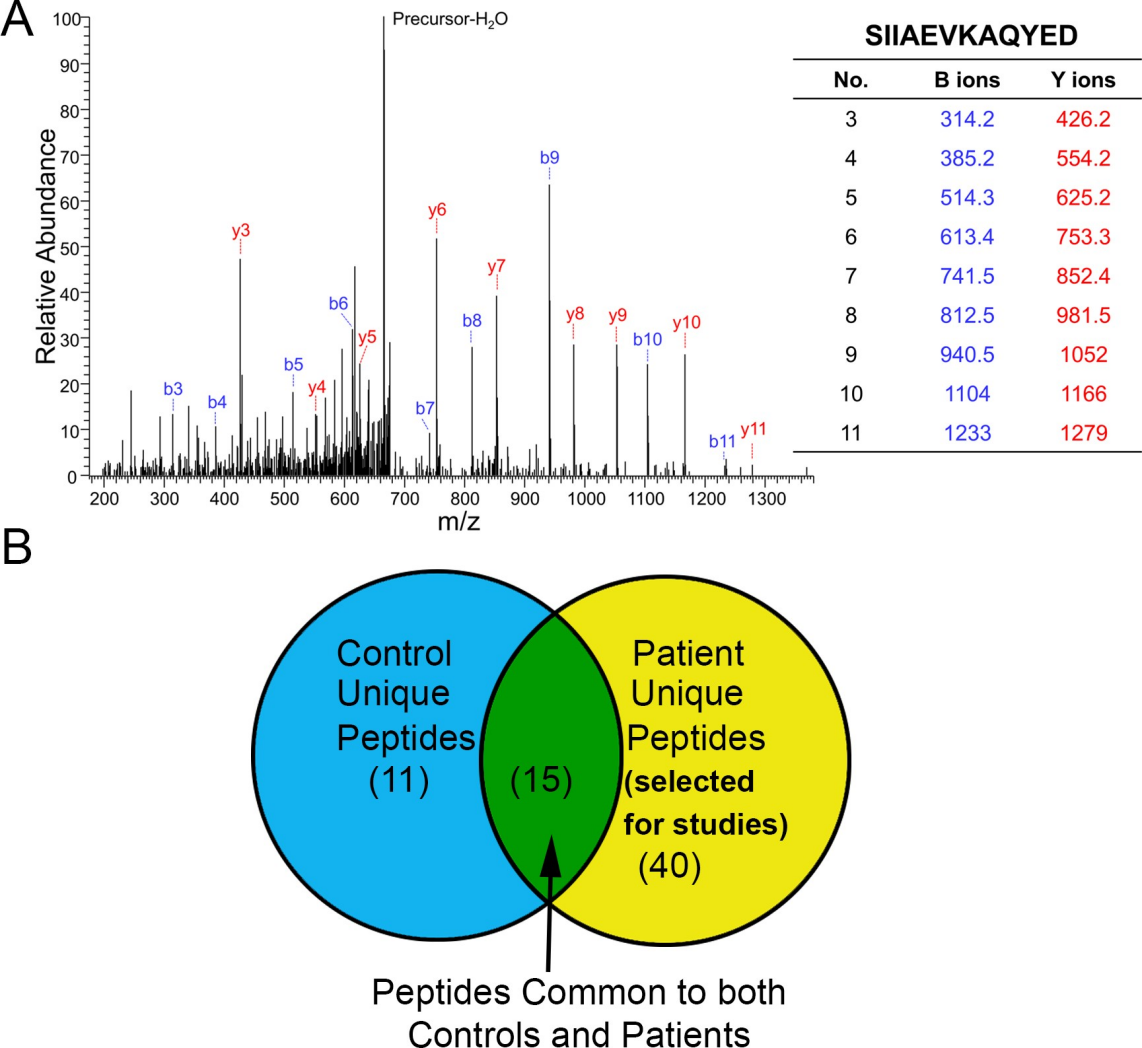
Peptide identification and selection. (A) Representative MS/MS spectrum of immuno-precipitated peptide, identified as Keratin, type II cytoskeletal 8. (B) Venn diagram depicting selection method for patient-unique peptides used in the study. Parenthesis indicates number of peptides detected.

UPLC-MS/MS analysis identified peptides eluted from the immune-precipitate (Fig 2A). The identified peptides were grouped according to the paradigm depicted in the Venn-diagram (Fig 2B). Peptides identified as unique to patients were further ranked according to frequency (Table 2). Of the 40 peptides found unique to the patients, 1 was common to 4/5 patients (Keratin 8), 1 was common to 3/5 (Bleomycin Hydrolase), and 7 were common to 2/5 [ARID1a, Desmocollin-1, Keratin 10 (2 peptides), Fibrinogen alpha, Keratin 1, and MEPCE]. The other proteins identified were unique to only single patient samples and ranked according to signal intensity (Table 2). The method thus identified novel self-antigens that may be potentially involved in CAD. The top 3 patient-unique proteins common among patients (Keratin 8, Bleomycin Hydrolase, and ARID1a) were selected for further study.

**Table 2:** Protein identification of peptides unique to patients.

**Unique to and Common among Patients Ranked by (Peptide Frequency)**
Keratin, type II cytoskeletal 8 (4/5)
Bleomycin hydrolase (3/5)
AT-rich interactive domain-containing protein 1A (2/5)
Desmocollin-1 (2/5)
Keratin, type I cytoskeletal 10 (2/5)
Keratin, type I cytoskeletal 10 (2/5)
Fibrinogen alpha chain (2/5)
Keratin, type II cytoskeletal 1 (2/5)
7SK snRNA methylphosphate capping enzyme (2/5)
**Unique to Individual Patients Ranked by Peptide Signal Intensity**
Twinkle protein, mitochondrial
Vacuolar protein sorting-associated protein 13C
Sal-like protein 3
Protein FAM83B
Protein S100-A8
Cystatin-M
Keratin, type I cytoskeletal 10
Keratin, type II cytoskeletal 1
Cardiomyopathy-associated protein 5
Keratin, type I cytoskeletal 10
Corneodesmosin
Keratin, type II cytoskeletal 1
Keratin, type II cytoskeletal 1
Spermatogenic leucine zipper protein 1
WD repeat-containing protein 72
Kinesin-like protein KIF15
Keratin, type I cytoskeletal 10
Corneodesmosin
G-protein coupled receptor 78
Kalirin
AF4/FMR2 family member 1
Vacuolar protein sorting-associated protein 18 homolog
Protein S100-A9
Putative E3 ubiquitin-protein ligase UNKL
Keratin, Type II cytoskeletal 1
DNA repair protein REV1
Keratin-associated protein 17–1
Keratin, type I cytoskeletal 9
Serine/threonine-protein kinase Sgk2
Putative heat shock protein HSP 90-beta 4
POU domain, class 6, transcription factor 1

### Mouse homolog search and peptide synthesis

Using the BLAST search tool, mouse homologs corresponding to the human peptides selected for further study were identified. The human Keratin, type II cytoskeletal 8 peptide sequence matched several isoforms of Keratin, type II in the mouse. The mouse isoform (Keratin 75) that was a 100% match of the identified human peptide was selected for further studies, called Keratin type II in this report given the highly conserved region of the matched peptide in Keratin type II isoforms. For the other peptides, the region of the homologous mouse protein that matched the human peptide sequence was selected for further study. Selected mouse peptides were flanked with amino-acids corresponding to the published sequence on both the carboxy- and amino- terminals (except Bleomycin Hydrolase because the homologous peptide is located at the amino-terminal) as described in Methods. The matching, flanked mouse peptides were synthesized at >95% purity and shown in Table 3. The flanked peptides were tested for MHC-I binding to mouse H2-D^b^ and H2-K^b^ using the *in silico* prediction tool by IEDB (Table 4).

**Table 3:** Sequence of mouse homologs of selected peptides flanked at terminals.

Peptide name	Peptide sequence
Keratin Type II	*MDNNRSLDLD*SIIAEVKAQYED*IANRSRAEAE*
Bleomycin Hydrolase	MNNAGLNSEKVS*ALIQKLNSDPQFVLAQNV*
ARID1a	*AQPSYQQQPQ*TQQPQLQS*SQPPYSQQPS*

Non-italicized segments are homologous to the human peptides identified by UPLC-MS/MS. Italicized segments correspond to the flanking sequence.

**Table 4:** Prediction scores for peptide binding to mouse MHC-I [29–33].

Allele	Start	End	Peptide	Method used	Percentile rank
			**Keratin Type II**		
H-2-Db	23	31	IANRSRAEA	Consensus (ann/comblib_sidney2008/smm)	3.5
H-2-Db	5	13	RSLDLDSII	Consensus (ann/comblib_sidney2008/smm)	4.7
H-2-Kb	5	12	RSLDLDSI	Consensus (ann/smm)	11.1
H-2-Db	13	21	IAEVKAQYE	Consensus (ann/comblib_sidney2008/smm)	17
H-2-Db	4	12	NRSLDLDSI	Consensus (ann/comblib_sidney2008/smm)	19
H-2-Kb	16	23	VKAQYEDI	Consensus (ann/smm)	19.5
			**Bleomycin Hydrolase**		
H-2-Db	3	11	NAGLNSEKV	Consensus (ann/comblib_sidney2008/smm)	0.9
H-2-Kb	11	18	VSALIQKL	Consensus (ann/smm)	2.45
H-2-Db	15	23	IQKLNSDPQ	Consensus (ann/comblib_sidney2008/smm)	3.4
H-2-Kb	23	30	QFVLAQNV	Consensus (ann/smm)	7.8
H-2-Db	22	30	PQFVLAQNV	Consensus (ann/comblib_sidney2008/smm)	15
H-2-Kb	7	14	NSEKVSAL	Consensus (ann/smm)	15.25
H-2-Db	12	20	SALIQKLNS	Consensus (ann/comblib_sidney2008/smm)	16
H-2-Db	7	15	NSEKVSALI	Consensus (ann/comblib_sidney2008/smm)	17
H-2-Db	17	25	KLNSDPQFV	Consensus (ann/comblib_sidney2008/smm)	17
			**ARID1a**		
H-2-Kb	19	26	SQPPYSQQ	Consensus (ann/smm)	13
H-2-Db	7	15	QQPQTQQPQ	Consensus (ann/comblib_sidney2008/smm)	17

### Immunologic memory to peptide antigens in mouse atherosclerosis

**Fig 3:**
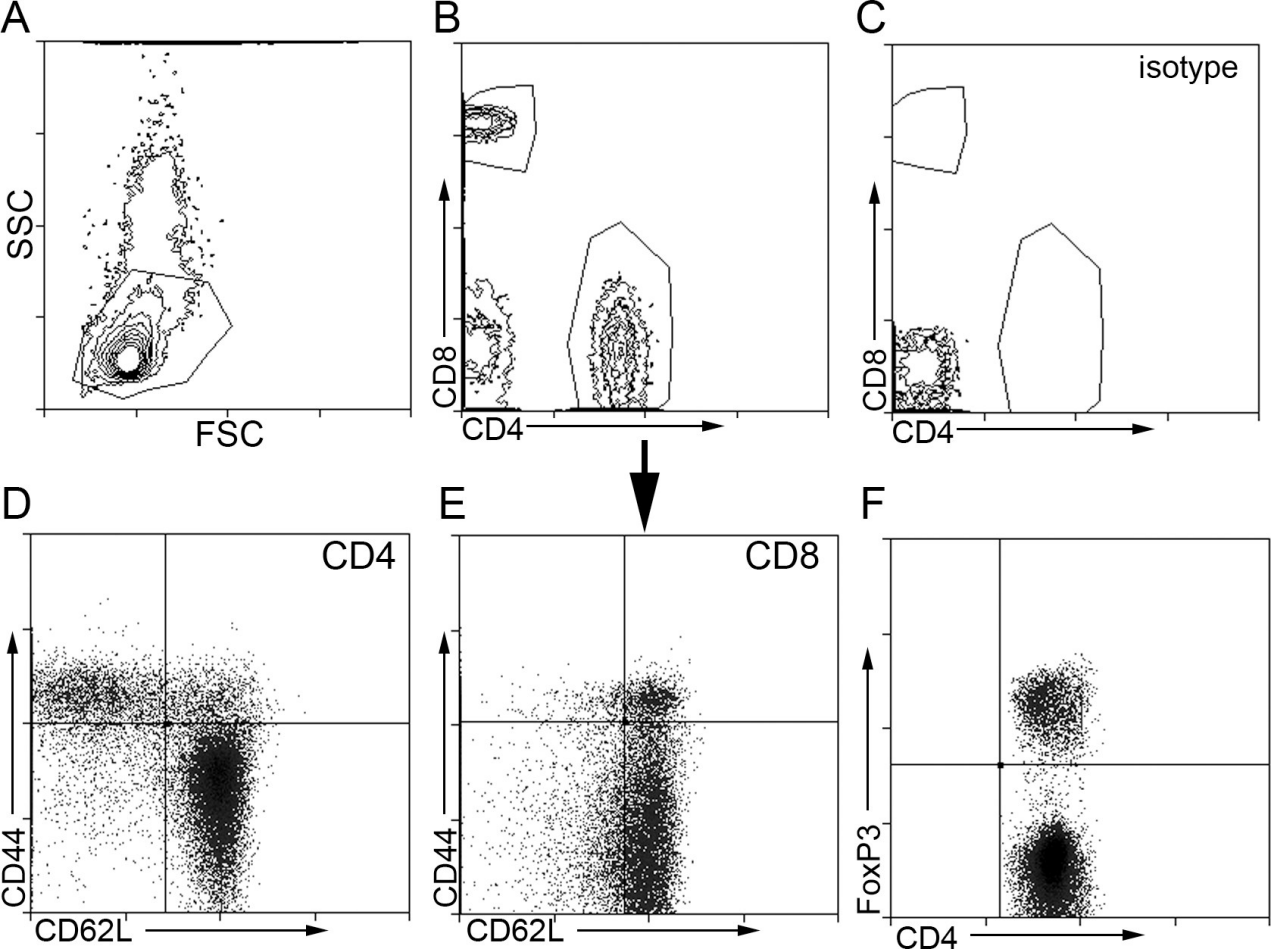
Gating strategy used for flow cytometry of splenocytes. Splenocytes were collected after 24-hour culture and stained for flow cytometry. Cell singlets were selected for analysis and non-viable cells were gated out. Size gating (A) was then performed to select CD8b+ or CD4+ cells (B). Isotype (C) was used as control. CD4+ or CD8b+ T cells (D and E, respectively) were then plotted on CD62L and CD44 scatter plots. GFP+ FoxP3+ cells (F) were plotted with the CD4+ T cell gate.

**Fig 4:**
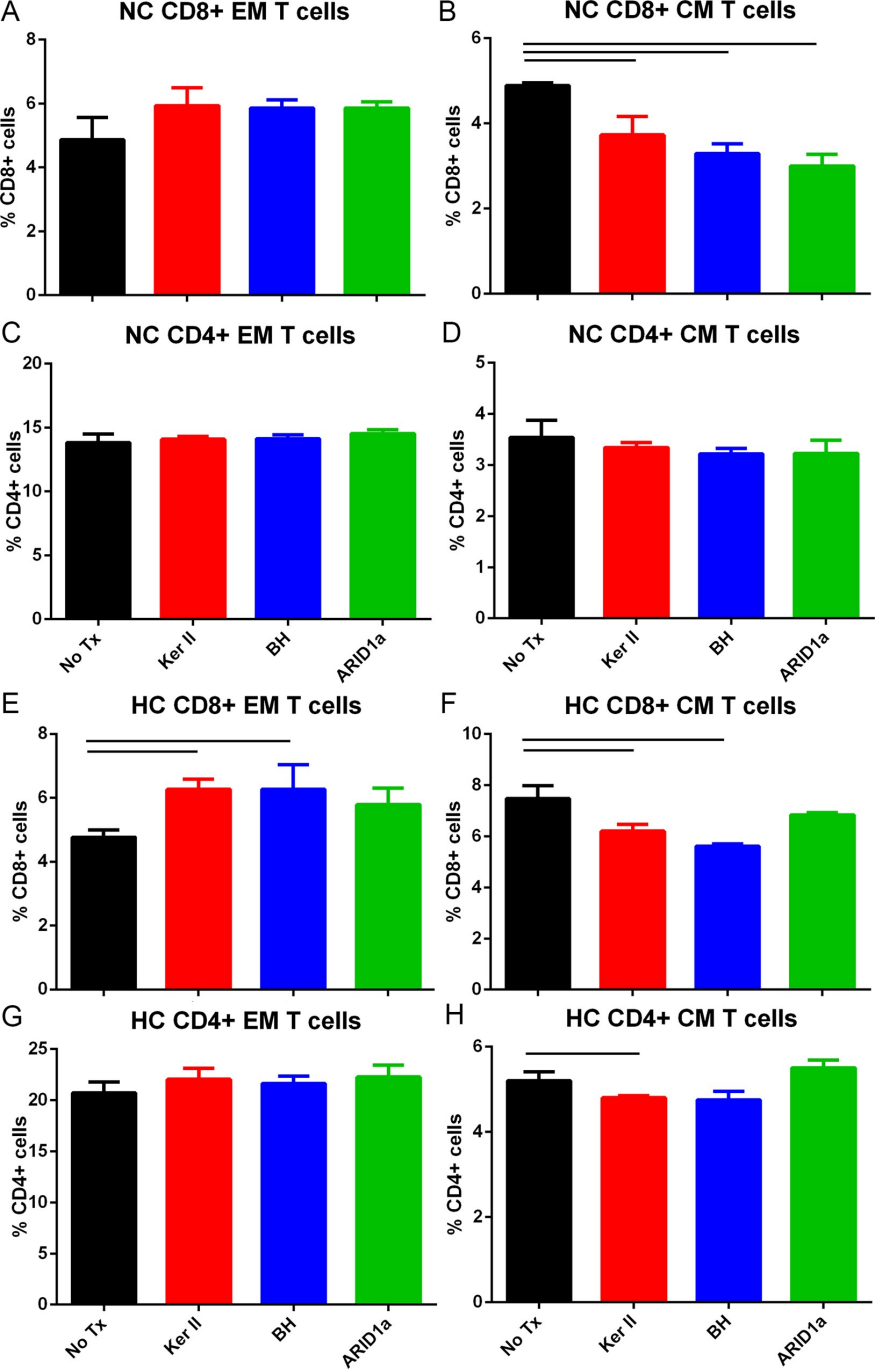
Peptide stimulation of splenocytes from apoE-/- mice fed normal chow or high fat diet. Splenocytes from male apoE-/- mice fed normal chow (NC, A-D) or high fat diet (HC, E-H) for 6 weeks were stimulated with 20μg/ml of individual peptides for 24 hours and assessed for CD44+CD62L(-) Effector Memory (EM) or CD44+CD62L+ Central Memory (CM) T cells. Gating strategy is as indicated in Fig 3. No Tx = no peptide treatment; Ker II = Keratin, type II; BH = Bleomycin Hydrolase. Bar over control and peptide treated column indicates P<0.05. Spleens from 2–3 mice were pooled per group in triplicates.

Given that atherosclerosis is the underlying condition that causes CAD, the potential for the identified peptides to be self-reactive antigens in atherosclerosis was tested using splenocytes from 13 week-old male apoE-/- mice fed normal chow. Male mice were used for the experiment since the peptides were identified using plasma from male patients. Gating strategy for fluorescent stains are depicted in Fig 3. There was no significant change in CD8+ Effector Memory (EM) T cells by any of the peptides tested (Fig 4A). On the other hand, CD8+ Central Memory (CM) T cells were significantly reduced by all the peptides tested (Fig 4B). The potential that some of the tested peptides may be reactive to MHC-II/CD4+ T cell signaling was also assessed. There was no significant effect on CD4+ EM T cells (Fig 4C) and CD4+ CM T cells (Fig 4D).

To assess the potential immunologic memory recall in an accelerated disease model, male apoE-/- mice were fed a high fat diet starting at 7 weeks of age for 6 weeks and the splenocytes subjected to peptide stimulation. CD8+ EM T cells were significantly increased by the peptides except for ARID1a (Fig 4E). CD8+ CM T cells were significantly reduced by the peptides except for ARID1a (Fig 4F). Similar to normal chow fed mice, the peptides again had no effect on CD4+ EM T cells (Fig 4G). CD4+ CM T cells were reduced to varying degrees by the peptides except ARID1a (Fig 4H). Thus, high fat diet fed mice had an enhanced immunologic response reactive to the self-antigens tested except ARID1a. The CD8+ T cell response to Keratin II from high fat diet fed mice is notable given that 4 out of 5 plasma samples from ACS patients had Keratin type II, cytoskeletal 8 detected as an immune-precipitated antigen (Table 2).

### Keratin II stimulates cytokine expression in T cells

**Fig 5:**
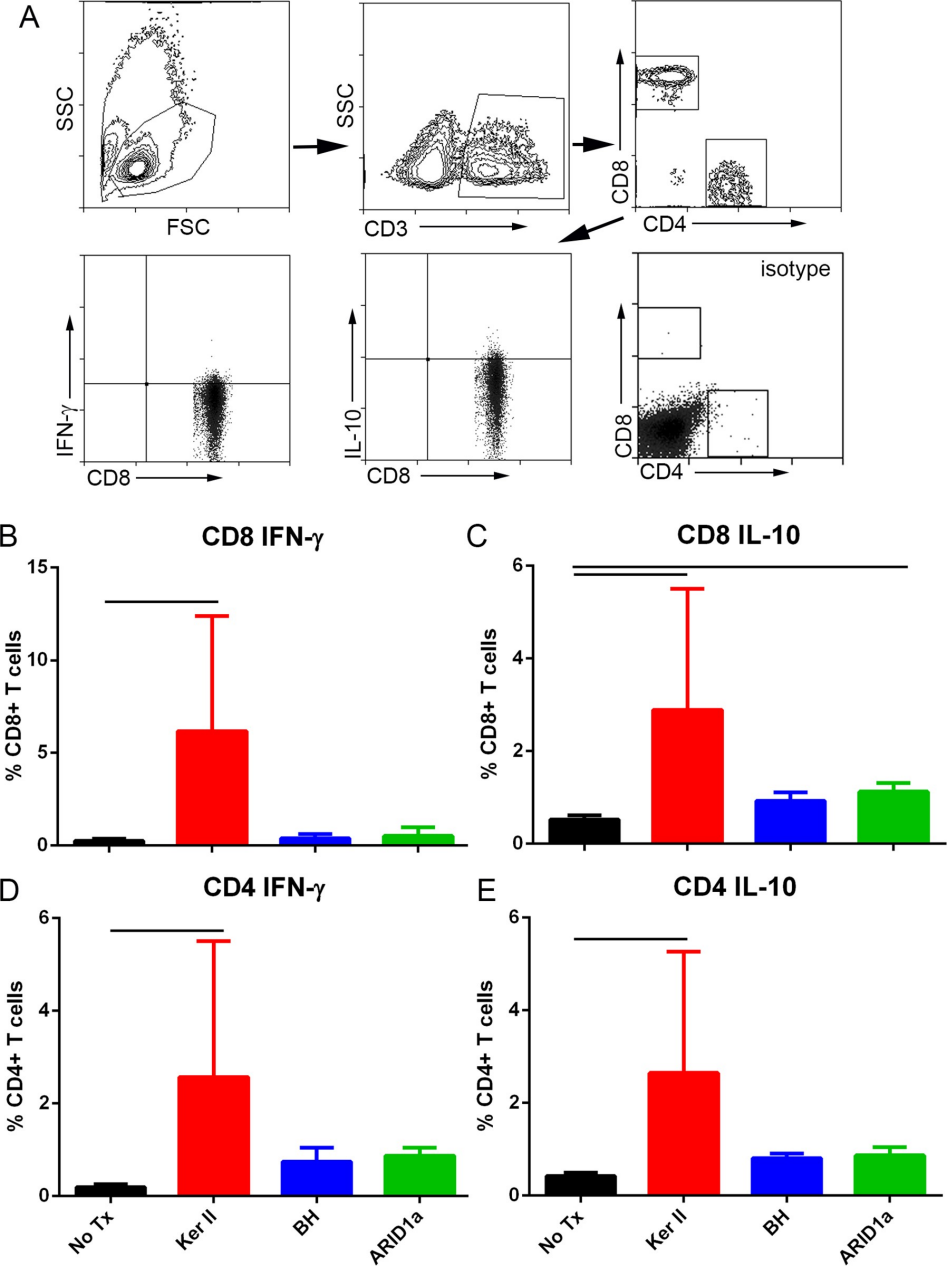
Cytokine expression of splenocytes from apoE-/- mice after peptide stimulation. Splenocytes were stimulated for 24 hours with 20μg/ml of the selected peptides to assess cytokine response. Gating depicted (A) includes cell singlets and excludes non-viable cells. Size-gated cells were then selected for CD3+ T cells, sub-grouped into CD8+ or CD4+ cells and assessed for IFN-γ or IL-10. No Tx = no peptide treatment; Ker II = Keratin, type II; BH = Bleomycin Hydrolase. Results are presented as percentage of CD8+ (B and C) or CD4+ (D and E) T cells. Spleens from 2–3 mice were pooled per group, (Ker II N = 3; No Tx, BH, ARID1a N = 4 each). Bar over columns indicate P<0.05.

Peptide stimulated splenocytes from apoE-/- mice were also assessed for intracellular cytokine expression. Keratin II peptide significantly increased T cells expressing IFN-γ and IL-10 in both CD8+ (Fig 5A–5C) and CD4+ (Fig 5D and 5E) T cells compared to non-stimulated cells. CD8+IL-10+ T cells were also increased by ARID1a peptide (Fig 5C).

### FoxP3 in the immune response to atherosclerosis antigens in mice

**Fig 6:**
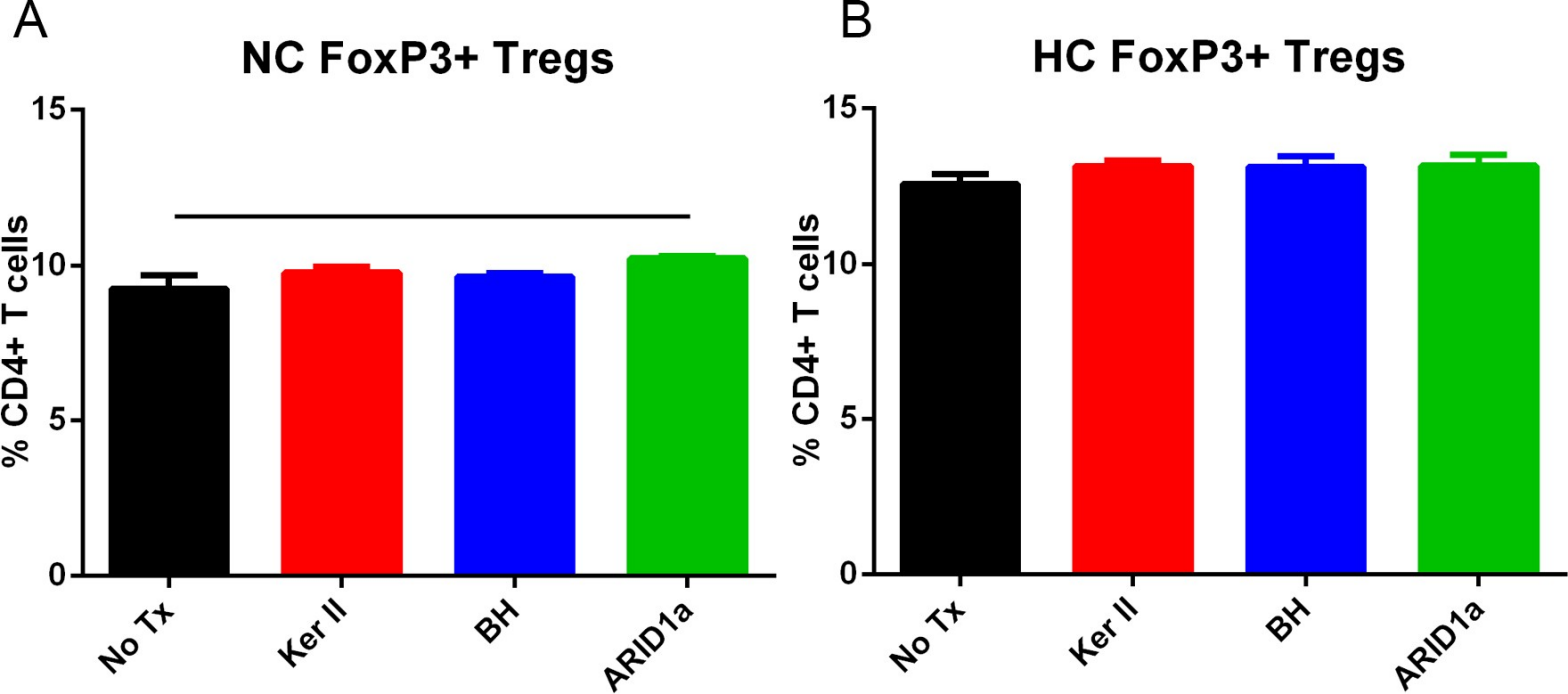
CD4+FoxP3+ T regulatory cells in splenocytes of apoE-/- mice fed normal chow or high fat diet. Splenocytes from apoE-/-FoxP3-GFP mice fed normal chow (NC; A) or high fat diet (HC; B) for 6 weeks were stimulated with 20μg/ml of individual peptides for 24 hours and assessed for FoxP3+ CD4+ T cells based on GFP fluorescence. No Tx = no peptide treatment; Ker II = Keratin, type II; BH = Bleomycin Hydrolase. Spleens from 2–3 mice were pooled per group, N = 3 each. Bar over columns indicate P<0.01.

Given that the self-reactive immune responses are modulated in part by FoxP3+ Tregs, we assessed CD4+FoxP3+ Tregs in peptide-stimulated splenocytes of mice fed normal or high fat diet. There was a small but significant increase in CD4+FoxP3+ Treg cells in splenocytes from mice fed normal chow stimulated with ARID1a (Fig 6A). No other differences were noted in splenocytes from both normal chow or high fat diet fed mice (Fig 6B) tested with any of the peptides, suggesting a limited peptide-specific FoxP3+ Treg response.

### Peptides do not provoke immune memory as myocardial infarction antigens

**Fig 7:**
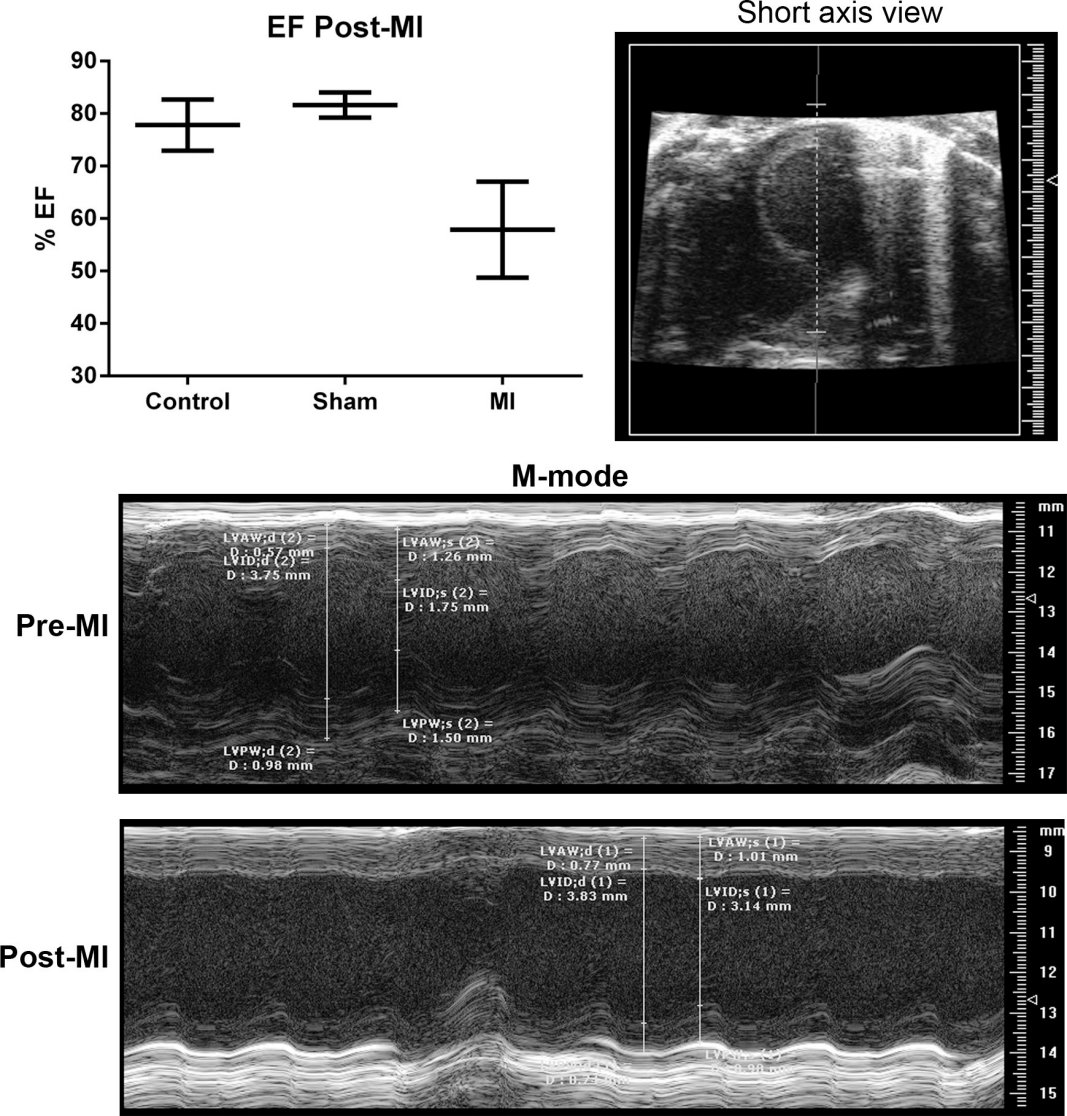
Myocardial infarction in apoE-/- mice. A subgroup of mice fed normal chow were subjected to surgical myocardial infarction (MI) at 13 weeks of age and euthanized 6 weeks later. Control mice were not subjected to surgical manipulation, sham mice had surgery without coronary artery occlusion. Change in ejection fraction at 6 weeks post-surgery confirmed MI. Representative echocardiographic recording is shown Pre-MI and Post-MI.

**Fig 8:**
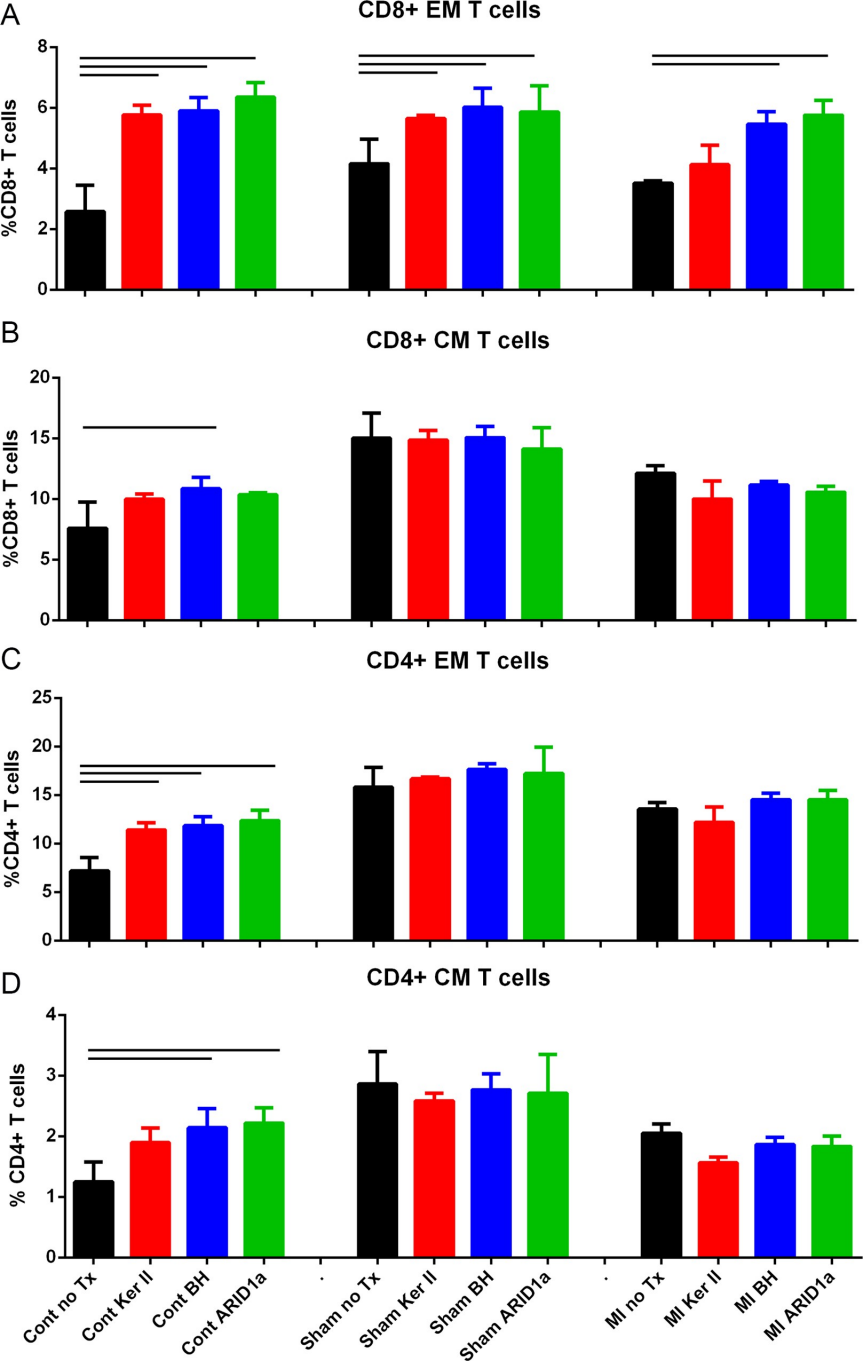
Peptide stimulation of splenocytes from apoE-/- mice subjected to MI. Splenocytes from male apoE-/- mice 6 weeks after MI were stimulated with individual peptides for 24 hours. Cells from mice not subjected to surgery (Cont) or subjected to sham surgery were compared to mice subjected to MI. Cells were harvested and stained for flow cytometry for CD8+ (A and B) or CD4+ (C and D) EM and CM T cells. Gating strategy is as indicated in Fig 3. Bar over control and peptide treated column indicates statistical significance within respective group (Control P<0.01; Sham P<0.05; MI P<0.01). Spleens from 2–3 mice were pooled per group, N = 3 each. No Tx = no peptide treatment; Ker II = Keratin, type II; BH = Bleomycin Hydrolase.

The peptides identified from the patients were presumed to be involved in the chronic inflammatory condition of CAD. However, the plasma samples were collected from patients diagnosed with myocardial infarction. Thus, it is possible that some of the self-antigens detected in the patient plasma were a consequence of the infarcted tissue. We therefore tested the immunologic response to peptide stimulation of splenocyte from male apoE-/- mice fed normal chow and subjected to surgical myocardial infarction at 13 weeks of age. Myocardial infarction was confirmed by change in ejection fraction as measured by echocardiography (Fig 7). Six weeks after MI, splenocytes were stimulated with the peptides. In control mice not subjected to surgical manipulation, all 3 peptides increased Effector Memory T cells (Fig 8A and 8C left column). In both sham and MI groups, CD8+ EM T cells were also increased by peptides except for Keratin II (Fig 8A, middle and right columns), which was non-responsive in the MI group. CD4+ T cell subtypes were unchanged in the sham and MI groups (Fig 8C and 8D, middle and right columns, respectively). The results suggested that MI itself did not increase self-reactive T cells in apoE-/- mice.

### Keratin 8 is a T cell reactive self-antigen in CAD patients

**Fig 9:**
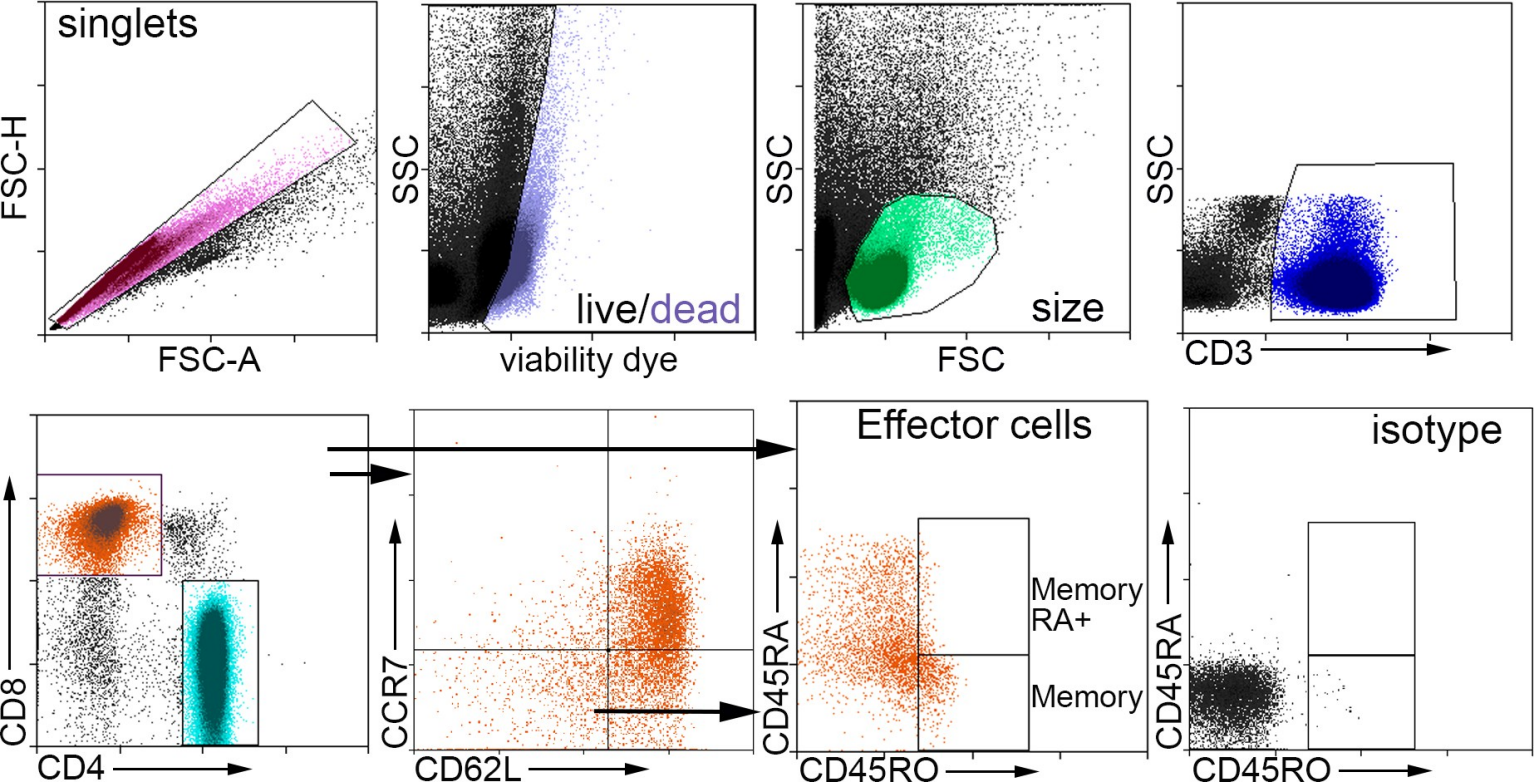
Gating strategy used for flow cytometry of human PBMCs. PBMCs were collected after 72 hours in culture with and without stimulation with Keratin 8 peptide. Positive control was stimulation with 0.5x T cell stimulation cocktail. Cells were collected and stained for flow cytometry. Cell singlets were selected and non-viable cells were excluded, followed by size-gating of viable cells. Cells were plotted on CD62L and CCR7 based on CD3+CD4+ cells or CD3+CD8+ cells. CD62L(-)CCR7(-) Effector cells were selected as T Effector Memory (TEM) or T Effector Memory RA+ (TEMRA) based on CD45RO/CD45RA stain.

**Fig 10:**
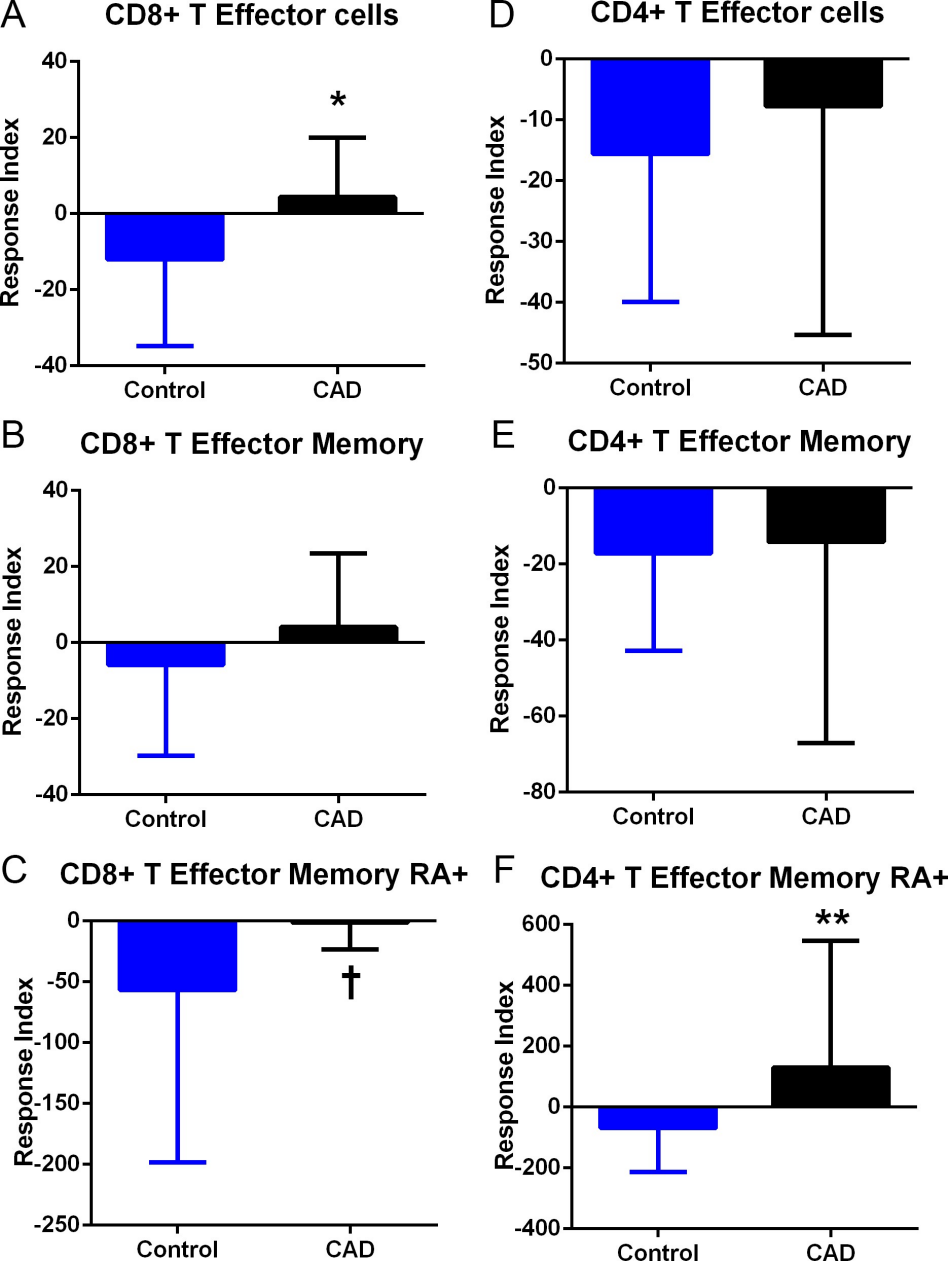
T Effector cell response to Keratin 8 peptide in CAD patient PBMC. CD8+ (A-C) and CD4+ (D-F) Effector T cells in patient PBMC compared to control PBMC. T Effector Memory (B and E) or T Effector Memory RA+ (C and F) cells were based on CD45RO/CD45RA stain as depicted in the gating strategy as described in Fig 9. *P<0.05; †P = 0.07; **P<0.01. Control N = 15; CAD N = 17.

**Fig 11:**
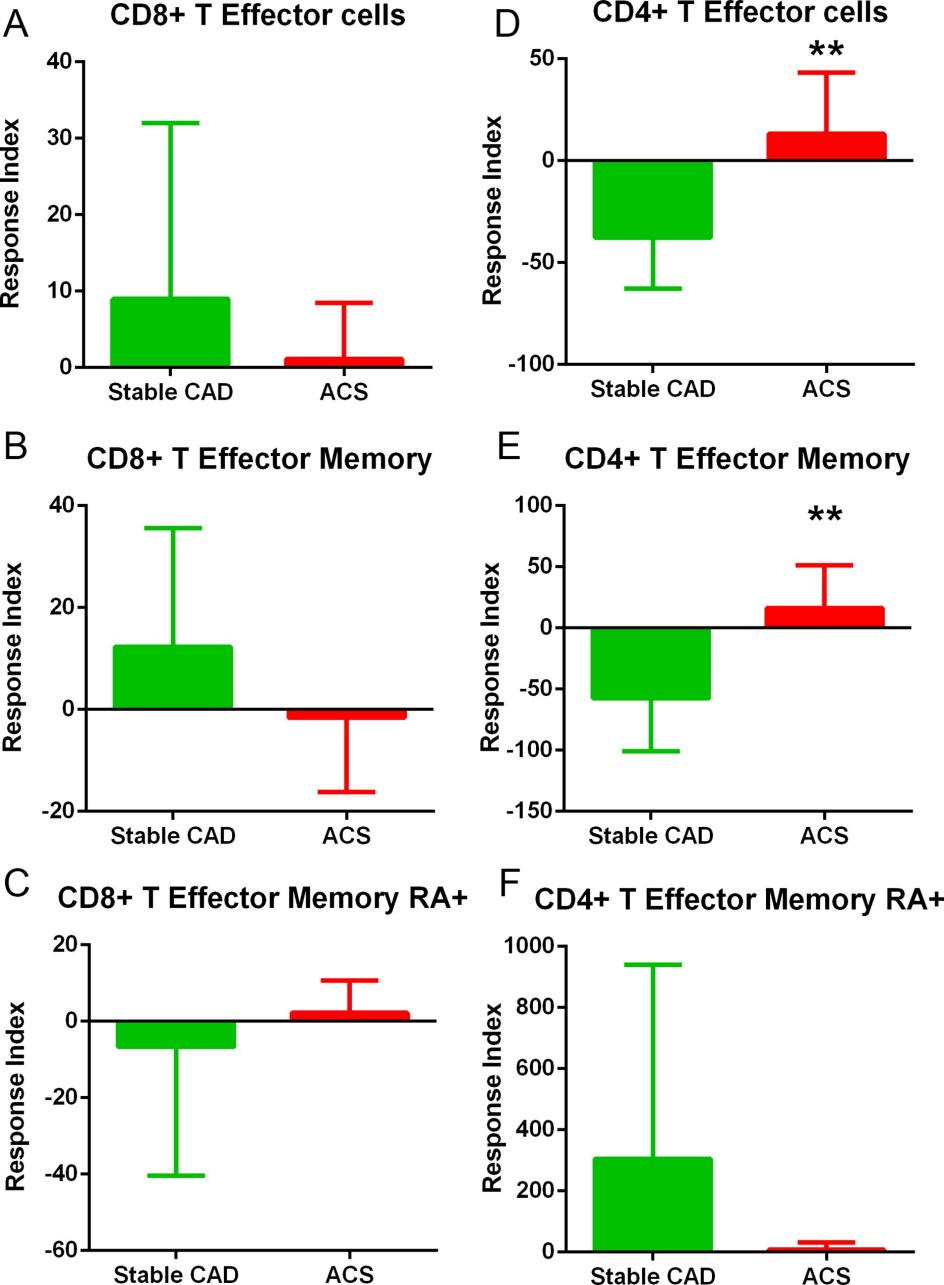
Effector T cell response in Stable CAD patients compared to ACS. CD8+ (A-C) and CD4+ (D-F) Effector T cells in Stable CAD patient PBMC compared to ACS patient PBMC. Gating strategy is as depicted in Fig 9. **P<0.01. Stable CAD N = 7; ACS N = 10.

Given that Keratin 8 was found in 4 out of 5 ACS plasma subjected to IP and UPLC-MS/MS, and the mouse peptide homolog induced a self-reactive Effector Memory T cell response in apoE-/- mice fed a high fat diet, we tested the translational potential of the findings by stimulating PBMC from CAD patients with the human Keratin 8 peptide. The peptide sequence from the IP and UPLC-MS/MS was flanked on the N- and C-terminals each with the corresponding 10 amino-acids (italicized) according to human Keratin 8 sequence: *MDNSRSLDMD*SIIAEVKAQYED*IANRSRAEAE*. The 32 amino-acid peptide is 94% homologous to the mouse sequence in Table 3. Cell gating for analysis is shown in Fig 9. T cells with Effector phenotype (CD45RO+) gated on CD62L(-)CCR7(-) were subtyped as T Effector Memory [CD45RA(-); TEM] or T Effector Memory RA+ (CD45RA+; TEMRA) [36–38]. CD8+ T Effector cell response to Keratin 8 was significantly increased in the CAD patient PBMCs compared to Controls (Fig 10A), with the difference found mainly in the TEMRA response (Fig 10B and 10C). CD4+ T Effector cell response was not significantly different between Controls and CAD patient PBMCs (Fig 10D and 10E), although increased TEMRA response in the CAD patients was observed (Fig 10F). To further characterize the response in CAD, patients were subclassified into Stable CAD or ACS. CD8+ T Effector cell responses were not significantly different between Stable CAD compared to ACS in any of the subtypes (Fig 11A–11C). CD4+ T Effector cell response was higher in ACS PBMCs compared to Stable CAD patients (Fig 11D) with the increase only in the TEM subtype (Fig 11E) but not in the TEMRA response (Fig 11F).

### Cytokine release in response to Keratin 8

ELISA using the conditioned medium from the PBMC culture did not show significant difference in IFN-γ or IL-10 levels (S1 Fig) after Keratin 8 peptide stimulation.

### PD-1 mRNA expression in Effector response to Keratin 8

**Fig 12:**
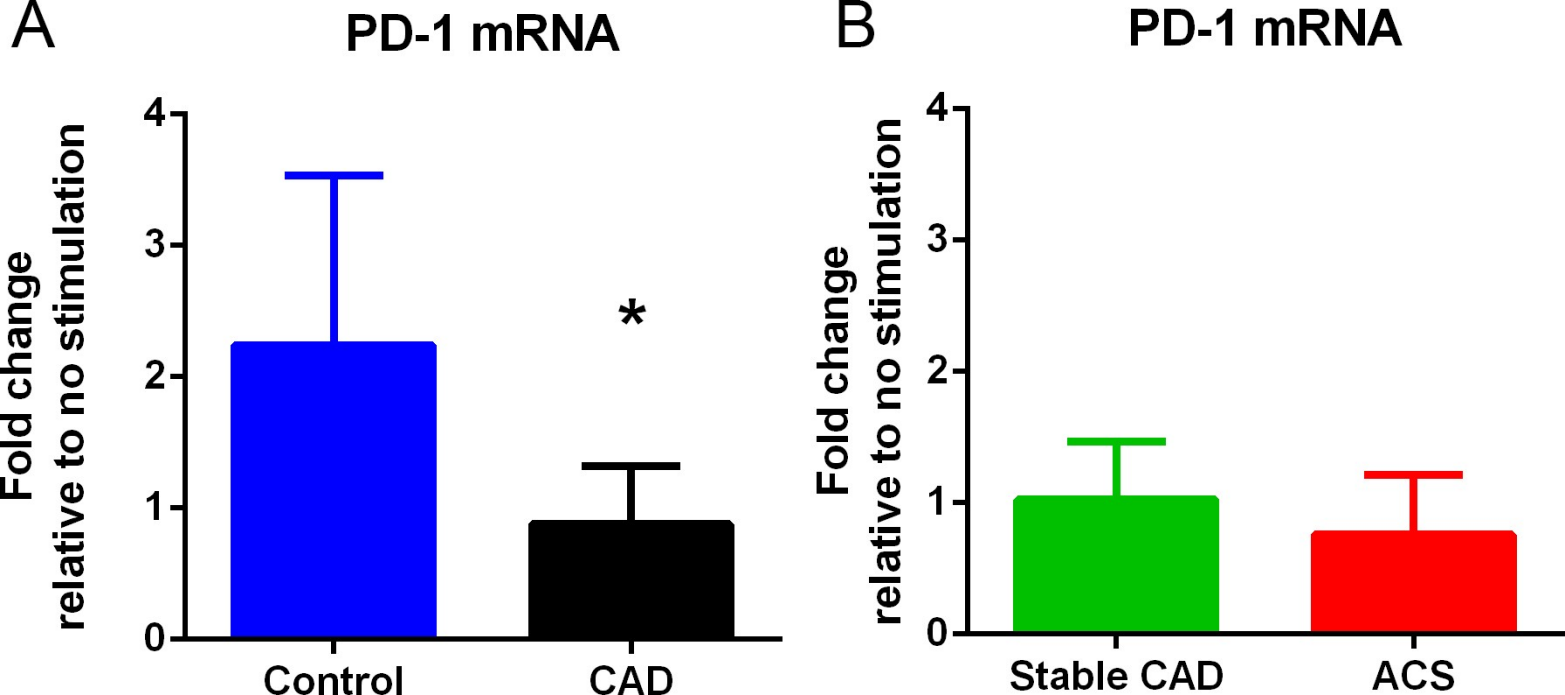
PBMC PD-1 mRNA expression in response to Keratin 8 stimulation. PD-1 mRNA expression (A) in Control PBMC (N = 5) compared to CAD patient PBMC (N = 9) *P<0.05. CAD patients sub grouped (B) as Stable CAD (N = 4) or ACS (N = 5).

PD-1 is an immune checkpoint protein expressed by activated T cells which functions to regulate immune homeostasis [39]. PBMCs from Controls stimulated with Keratin 8 peptide significantly increased PD-1 mRNA expression but was unchanged in CAD (Fig 12A). Stable CAD and ACS had comparable PD-1 mRNA expression (Fig 12B). The results suggest that a lack of increase in PD-1 expression may be involved in immune self-reactivity to the self-antigen Keratin 8 in CAD.

## Discussion

We report on novel self-antigens potentially involved in atherosclerosis using IP of soluble MHC-I/peptide complexes from CAD patient plasma and MS/MS to identify specific peptides. Several generalizations can be made based on our results: 1) Soluble MHC-I/peptide complexes from CAD patients can be used to generate immunopeptidomic profiles of the underlying disease, evidenced by the novel self-antigens described; 2) Feeding of mice with high fat diet increased the propensity for memory T cells to respond to the self-peptides tested; 3) The peptides identified appear to be of more relevance to a response to atherosclerosis rather than a response to MI; and 4) CAD patients have T cells reactive to Keratin 8 peptide.

The first major observation in our report is that there is some degree of uniqueness to the soluble MHC-I/peptide complexes that are present in the plasma of CAD patients compared to controls, consistent with our hypothesis. As a discovery cohort, we selected only 5 ACS patient plasma samples and 5 controls. ACS plasma samples were used because these patients categorically have CAD. Males were selected for this cohort to limit potential effects of known sexual dimorphism in immune responses [40]. We identified several peptides unique to, and common among, patients. We then tested if the mouse homologs of the peptides provoked immune responses during the disease process in male apoE-/- mice to assess functional relevance to atherosclerosis, the underlying cause of CAD.

Effector memory (EM) T cells correlate with disease severity in mouse models of atherosclerosis [6], altered by high fat diet [19,35] and are markers for screening of immune responses in atherosclerosis [41]. There was a lack of responsiveness to the selected peptides in EM T cells from apoE-/- mice fed normal chow, although there was a degree of suppression in the CM T cell population, suggesting tolerance to the peptides. The involvement of CD4+ Treg cells in this response appears to be limited to ARID1a. On the other hand, there was significantly increased CD8+ EM T cells after peptide stimulation except for ARID1a when apoE-/- mice were fed a high fat diet. The results suggest that the selected peptides may be involved in the immune response to atherosclerosis, which in the apoE-/- model is accelerated by high fat diet. Of particular interest was the response to Keratin II stimulation by T cells from the high fat diet fed mice which was detected as a potential self-antigen in majority of the patient plasma samples tested.

We also observed increased T cells expressing IFN-γ and IL-10 in splenocytes stimulated with Keratin II. Although suggestive of immune activation in mice, clinical studies suggest that immune phenotyping using T Effector Memory cells is more sensitive and correlates with different stages of CAD as compared to cytokine expression [6,22]. Complex and ruptured plaques from CAD patients had increased presence of T Effector Memory cells, whereas the cells decreased in healed plaques [22]. Reports also suggest a heterogenous population of T cells with a lack of cytokine bias in plaques [22,42]. Thus, the significance of increased mouse T cell cytokine expression in the context of CAD in humans remains to be determined [43].

Our original hypothesis was that the soluble MHC-I/peptide complexes that would be immuno-precipitated would be potential CAD antigens. However, there was the possibility that the antigens would be a response to injured myocardium since the patient cohort used was from a prior study (AZACS) wherein patients enrolled were diagnosed with ACS [23]. Thus, we assessed whether apoE-/- mice subjected to surgically-induced MI would have immunologic memory to the peptide antigens. Mice were left to recover for 6 weeks post-MI in order for the immune system to generate memory cells in response to neo-antigens caused by infarcted cardiac tissue [44].

Surgical manipulation in male mice induced a generalized non-responsiveness in CD8+ CM T cells and both CD4+ memory T cell subtypes, suggesting that surgical trauma has a degree of anergic effect on immune responses to self-antigens in apoE-/- mice. Thus, our results show that the self-peptides that were identified are potentially involved in response to atherosclerosis and not in response to neo-antigens from myocardial tissue damage after MI [44].

Our results implicated self-antigens in the atherosclerotic disease process of apoE-/- mice. However, relevance to clinical disease remained unclear and translational experiments needed to be performed. The determination of which self-antigen to test was based in part on the nature of the protein sources of the self-antigens.

Bleomycin Hydrolase is the enzyme that mediates protection from bleomycin toxicity. Neonatal survival of knockout mice is 65% and surviving mice have tail dermatitis [45]. Bleomycin Hydrolase protects against homocysteine toxicity by efficient homocysteine thiolactonase activity [46], has cysteine protease activity that is involved in peptide trimming for MHC-I presentation [47], and is expressed by inflammatory cells of human atherosclerotic lesions [48]. Interestingly, dysregulated expression is observed in psoriasis patients [49], who are at a higher risk of cardiovascular events [17].

Adenine-thymine (AT)–rich interactive domain-containing protein 1a (ARID1a) is a tumor suppressor gene. Also called BAF250a, it is part of the BAF complex that regulates chromatin structure [50]. Its mutation rate is associated with Clear Cell Renal Cell Carcinoma [51] and loss of expression was observed in a study of early stage colorectal cancer [52].

Keratins are intermediate filament proteins and have been associated with autoimmunity, specifically rheumatoid arthritis [53]. It has been suggested that keratins are mimotopes of the MHC-I haplotype HLA-B27 which is implicated in several autoimmune diseases [54,55]. Keratin 8 is found in atherosclerotic plaques [56], neointimal and synthetic smooth muscle cells [57,58], and saphenous vein grafts [59], and its ectopic expression has been linked to heart failure [60]. Keratin 8 was reported as an autoantigen in uveitis [54,55] and in rheumatoid arthritis [61] and may be involved in immune escape mechanisms by cancer cells [62]. An intriguing report is the function attributed to Keratin 8 that limits IL-1β mediated inflammatory signaling [63] indicating its importance in the inflammasome pathway of innate immunity, the relevance of which was highlighted by the CANTOS study [2]. Thus, Keratin 8 is functionally involved in both innate and adaptive immune responses. Given that Keratin 8 was found in 4 out of 5 plasma samples from ACS patients in the immune-peptidome analysis, provoked memory T cell response in high fat diet fed apoE-/- mice, and is functionally involved in immune responses based on citations discussed above, it was selected for translational studies using PBMCs collected from a spectrum of CAD patients. We selected markers of T Effector Memory cells to characterize the response to Keratin 8 given the reported sensitivity of these T cell subpopulations associated with CAD [6,22]. The results were expressed as Response Index given the potential variability inherent in primary cells cultured over time in vitro, especially that from the CAD patients.

Significantly different CD8+ T Effector response in Controls compared to CAD patients was observed after stimulation with Keratin 8 peptide, mainly attributable to reduced TEMRA cells in Controls. This suggests that immune regulation in response to Keratin 8 peptide is altered in CAD patients. CD4+ TEMRA response to Keratin 8 peptide was increased in CAD patients suggesting that self-reactive T Effector Memory cells are both CD8+ and CD4+ subtypes. T Effector responses in CAD patients in our study were heterogenous, with notable differences especially in the CD4+ T cell population of ACS patients compared to Stable CAD. The significance of these results remain to be investigated but are in agreement with the report that showed TEM cells as independent predictor of carotid intima-media thickness in CAD patients [6]. The same report showed significantly increased TEM cells in chronic stable angina and acute myocardial infarction patients [6]. Their report used T cell markers that focused on TEM cells [6], while our study further distinguished TEM from TEMRA cells after peptide stimulation [36,37]. TEMRA cells are highly differentiated memory cells with rapid effector responses in humans [38]. Although initially observed in CD8+ T cells, CD4+ TEMRA have also been reported [64]. The precise role of TEM in CAD remains to be clarified but their presence in plaques has been known for some time [65]. Increased TEM cells in plaques are associated with increased plaque progression, with the highest presence reported in thin-cap fibroatheromas and ruptured plaques [22]. Our results extend the cited reports on TEM cells in CAD [6,22] by using a specific self-peptide to stimulate PBMCs to assess TEM cell responses.

Although soluble MHC-I/peptide complexes were used to identify potential self-antigens, it is unlikely that the self-reactive response would be restricted to CD8+ T cells. Interaction and cooperation between CD4+ and CD8+ T cell immune responses are reported in depth [66,67]. This is further reflected in the subgroup analysis of the CAD response, where significant difference was observed in the CD4+ T Effector cell response in ACS compared to Stable CAD, mainly in the TEM cell subtype.

The first inclination would be to disregard Keratin 8 as a contaminant of the IP UPLC-MS/MS process. However, in the context of other reports cited above combined with our results, it is likely that Keratin 8 is involved as a self-antigen in cardiovascular disease. Keratin 8 interacts with HLA class-I [62], and a 10-mer Keratin 8 peptide was shown to bind with high affinity to HLA-A*0201 and several 9 to11-mer Keratin 8 peptides had moderate affinity for HLA-A*1101 [68]. In the same report, several other Keratin 8 peptides were predicted to bind with similar affinity to these HLA-A alleles [68]. The normal response to Keratin 8 peptides would be tolerance, supported by the report that it is expressed in the thymus and lymph nodes of mice [69]. Expression of antigens in the thymus and lymph nodes is generally known to induce tolerance. The immune checkpoint PD-1 regulates immune homeostasis and is involved in immune tolerance. It is a therapeutic target in immune-oncology to circumvent tumor-induced overexpression of PD-1L that renders tumor cells tolerant to immune surveillance [39]. The lack of increase in PD-1 mRNA expression by Keratin 8-stimulated PBMCs from CAD patients suggests an aberration of normal tolerance pathways to the self-antigen. Aberrant self-reactive immune responses to Keratin 8 in the context of CAD needs further investigation but its potential involvement is supported by the report that Keratin 8 binds to small dense, low-density lipoprotein (sdLDL) [70]. Analysis of over 11,000 subjects in the Atherosclerosis Risk in Communitites (ARIC) study showed that sdLDL concentration predicted risk for coronary heart disease [71].

There are limitations in our study, including the small number of patient plasma used for the immunopeptidomic discovery cohort. Although the number of subjects used for PBMC stimulation was also small, it provided corroborating evidence of T cell immune response to one specific self-antigen–Keratin 8 in CAD patients suggesting that the approach is sound. Our study suggests feasibility of the concept, providing reasonable evidence to further investigate the Keratin 8 peptide as a self-antigen in a bigger CAD cohort for validation. This is important given that the Keratin 8 peptide may have provoked immune responses specific to CAD but not necessarily specific to Keratin 8 peptide itself. This can only be addressed in a validation study with a wider scope that includes methods to test antigen presentation by patient PBMCs. Although the use of the mouse model to assess the potential role of the peptides as self-antigens may be viewed as another limitation, we needed to first determine feasibility, and this could only be reasonably tested in an animal model of atherosclerosis. On the other hand, the mouse model provides a potential opportunity to investigate in depth mechanistic pathways of self-antigen involvement in atherosclerosis, which can be achieved with immunization studies.

Translational experiments with the Keratin 8 peptide in patient PBMCs were performed after verification of its potential role in the mouse disease model. More studies need to be performed to generate a library of CAD immune peptidome in order that translational tests of human immune responses in vitro will be more productive. In particular, a study with a wider scope to include female CAD immune peptidome profiling is necessary to investigate potential sex-related immune functions [40,72,73].

In conclusion, we have identified several novel peptides unique to CAD patients that may be involved in the self-reactive immune response to atherosclerosis using an immunopeptidomic approach. We provide proof-of-concept as well as a road map to discover and test potentially relevant and novel immune antigens involved in CAD. Translational potential of the studies is affirmed by the finding that at least one of the identified self-antigens, Keratin 8, provoked a self-reactive T cell response suggesting its involvement in the immune-inflammatory mechanism of CAD [2]. Our report facilitates the methodical discovery of novel pathways of immune activation and regulation in the context of inflammatory signaling in CAD.

## Supporting information

S1 Table Protein identification of peptides unique to controls. (PDF)

S2 Table Protein identification of peptides common to controls and patients. (PDF)

S1 Fig Cytokine release in conditioned medium of human PBMCs. ELISA for IFN-γ and IL-10 using conditioned medium collected from the 72-hour culture of PBMCs from Control and CAD patients. NS = non-stimulated; St = stimulation with Keratin 8 peptide.
(PDF)
