# Supplementary material for: Keratin 8 is a potential self-antigen in the coronary artery disease immunopeptidome: A translational approach
Source: PLoS One. 2019 Feb 27;14(2):e0213025. doi: 10.1371/journal.pone.0213025 (PMC6392305; doi:10.1371/journal.pone.0213025)
Supplement: S1 Table — (PDF) [file pone.0213025.s001.pdf]

S1 Table. Protein identification of peptides unique to controls.

Calmodulin-like protein 5
Mitochondrial dynamic protein MID49
Scavenger receptor cysteine-rich type 1 protein M160
Heterogeneous nuclear ribonucleoprotein R
Leucine-rich repeats and immunoglobulin-like domains protein 3
Filaggrin
Polycomb group RING finger protein 2;Polycomb complex protein BMI-1
Heat shock 70 kDa protein 14
AT-rich interactive domain-containing protein 1B
Equilibrative nucleoside transporter 4
Filaggrin
