# Supplementary material for: Keratin 8 is a potential self-antigen in the coronary artery disease immunopeptidome: A translational approach
Source: PLoS One. 2019 Feb 27;14(2):e0213025. doi: 10.1371/journal.pone.0213025 (PMC6392305; doi:10.1371/journal.pone.0213025)
Supplement: S2 Table — (PDF) [file pone.0213025.s002.pdf]

**S2 Table. Protein identification of peptides common to controls and patients.**

| |
|---|
| Keratin, type II cytoskeletal 1 |
| Keratin, type II cytoskeletal 6A;Keratin, type II cytoskeletal 6B;Keratin, type II cytoskeletal 6C |
| Keratin, type II cytoskeletal 6A;Keratin, type II cytoskeletal 6B;Keratin, type II cytoskeletal 6C |
| Nesprin-2 |
| Corneodesmosin |
| Integrin beta-1;Integrin beta |
| 60S ribosomal protein L22 |
| Keratin, type II cytoskeletal 6A;Keratin, type II cytoskeletal 6B;Keratin, type II cytoskeletal 6C |
| Ligand-dependent nuclear receptor-interacting factor 1 |
| Keratin, type II cytoskeletal 73;Keratin, type II cytoskeletal 74;Keratin, type II cytoskeletal 71 |
| Keratin, type II cytoskeletal 2 epidermal |
| RNA exonuclease 1 homolog;Exonuclease GOR |
| Coiled-coil domain-containing protein 30 |
| Keratin, type II cytoskeletal 6A;Keratin, type II cytoskeletal 6B;Keratin, type II cytoskeletal 6C |
| Keratin, type I cytoskeletal 9 |
