# Supplementary figures and images for: Keratin 8 is a potential self-antigen in the coronary artery disease immunopeptidome: A translational approach
Source: PLoS One. 2019 Feb 27;14(2):e0213025. doi: 10.1371/journal.pone.0213025 (PMC6392305; doi:10.1371/journal.pone.0213025)

S1 Fig: Cytokine release in conditioned medium of human PBMCs.

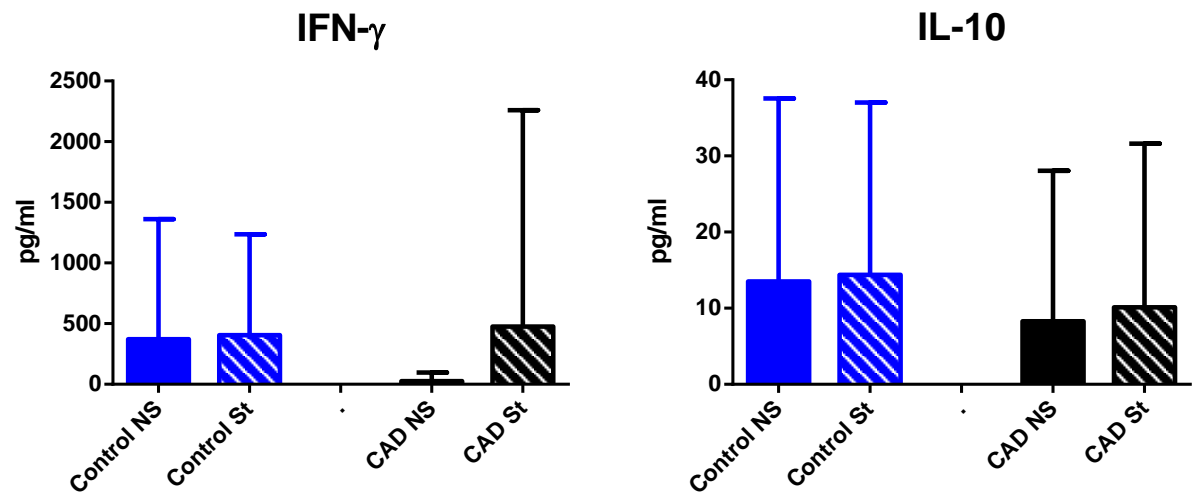

Supplement: S1 Fig — ELISA for IFN-γ and IL-10 using conditioned medium collected from the 72-hour culture of PBMCs from Control and CAD patients. NS = non-stimulated; St = stimulation with Keratin 8 peptide. (PDF) [file pone.0213025.s003.pdf]
